# The Journey to Adulthood: A Systematic Review of Interventions in Type 1 Diabetes Paediatric to Adult Transition Care

**DOI:** 10.1155/2024/1773726

**Published:** 2024-09-26

**Authors:** Nada Aljohani, Sara Donetto, Mette Due-Christensen, Angus Forbes

**Affiliations:** ^1^ Department of Clinical Research in Diabetes Division of Care in Long-term Conditions Florence Nightingale Faculty of Nursing, Midwifery and Palliative Care King's College London, London, UK; ^2^ Department of Medical and Surgical Nursing College of Nursing Princess Nourah bint Abdulrahman University, Riyadh, Saudi Arabia; ^3^ Department of Health Promotion Steno Diabetes Center Copenhagen, Region Hovedstaden, Denmark

**Keywords:** adolescence, adulthood, paediatric, T1DM, transition, type 1 diabetes

## Abstract

Young people with type 1 diabetes mellitus (T1DM) transition from paediatric to adult services when they reach late adolescence. This can be a risky period for young people, and it has been associated with a deterioration in glycaemic control and disengagement from diabetes services. This review aimed to identify current interventions addressing the following questions: What adolescents with T1DM healthcare transition interventions have been evaluated? What are the underlying theories and components of these interventions? What outcomes have been considered in these evaluations? Databases, trial registries and other sources were searched using the population and intervention keywords. Studies were included if they explicitly reported a transition intervention targeting young people aged 10–25 years. Studies were critically apprised, and data were extracted. Both tabular and narrative data synthesis were used. The review included 22 studies. Most interventions were service-oriented, with little use of theory. The interventions included transition planning, service coordination, pre-transition education, transition clinics, prompting strategies and other less frequent components. Most studies reported metabolic outcomes, with limited data on psychological outcomes such as diabetes adaptation, acceptance and self-management activation. It is inconsistent how each outcome was defined, measured or reported. Consequently, effective theory-based interventional transition models are yet to be identified.

## 1. Background

The transition from paediatric to adult healthcare can be challenging for young people with type 1 diabetes mellitus (T1DM) as it occurs during adolescence, which is a period characterised by significant physiological, psychological and social development [1, 2]. Hence, transitional care involves more than relocating young people from paediatric to adult services; it also needs to support their growing autonomy in managing their condition [3]. The transition also involves a change in the model of care they experience, where paediatric care tends to focus on the family unit and involves parents in decision-making; adult care assumes that the person with diabetes has the capacity for independent self-management and self-advocacy [4]. As each young person's psychological and social maturity varies alongside variable parental dynamics, transition care needs some level of individualisation. Socio-cultural expectations are also relevant to the transition experiences of young people, as different social norms govern definitions of adulthood in terms of age, roles and responsibilities [5].

Despite much innovation in transition care and a growing recognition of its importance, the transition period is still associated with reduced care engagement and self-management activation [1, 6, 7], with corresponding increases in hyperglycaemia and the risk of diabetes complications [8]. A third of young people with T1DM disengage from diabetes services during this time [9], and nearly half of them experience transitional difficulties, such as processing change, difficulties in developing autonomy in or engagement with their self-management, and dissatisfaction with the adult-oriented care model [8]. Incident cases of diabetic ketoacidosis (DKA) also are higher post-transition [10]. Therefore, developing effective strategies to improve care activation and reduce adverse events among young people during the transition remains a high priority. Effective transition care may also help reduce the risk of future diabetes complications and enhance the physical, mental and social well-being of young people with T1DM [11]. It is recognised that improving transition outcomes requires multimodal strategies addressing both the transition and the developmental context of the young person [9, 12]. Such strategies also need to integrate care in relation to multiple professionals involved in the transition from both adult and paediatric services, as well as the young person and their family. The challenge is in identifying the care processes and strategies that can best address these complex problems. This review provides a comprehensive perspective on transition interventions than reported in previous reviews of this topic [13–15]. While previous reviews have examined the nature and effectiveness of current intervention models, this review goes beyond that by also considering and explicating the underpinning theories, mechanisms, ‘active ingredients', and delivery methods of these interventions. The aim of this review is to map and explore the underlying theories and components of interventions implemented to facilitate and support adolescents with T1DM as they transition from paediatric to adult healthcare. By incorporating a broader range of factors, this review offers a more in-depth understanding of transition interventions for adolescents and young adults with chronic health conditions and disabilities. The inclusion of these additional elements enhances the overall credibility and reliability of the findings, making this review a valuable resource for researchers, practitioners, and policymakers in the field of transition interventions.

## 2. Methods

A systematic review of studies designed to evaluate transition interventions was performed in accordance with the Joanna Briggs Institute's (JBI) guidelines for systematic reviews [16]. In alignment with the theory-building stage of the UK Medical Research Council (MRC) complex interventions evaluation framework, the review's focus was on developing a contextualised theoretical understanding of how the existing intervention components mediate transition experiences and outcomes [17].The review aimed to map and synthesise the underpinning theories and components of previous interventions used to facilitate and support adolescents with T1DM in the transition from paediatric to adult healthcare in order to explicate a better understanding of the active mechanisms in these interventions and their impact on care outcomes. The review objectives were as follows:

• To identify studies evaluating transition interventions for adolescents with T1DM.
• To critically appraise the identified studies.
• To extract relevant data in relation to the intervention content, context and study outcomes.
• To undertake a tabulated and narrative synthesis of the study findings.
• To explicate a theoretical model integrating intervention content, mechanisms and outcomes.

Given the heterogeneous nature and complexity of transition interventions and study design, a preliminary scoping search was undertaken to inform the review design and methods. The search showed that a few studies included other long-term conditions (these studies were included if data exclusive to people with T1DM were reported) or the proportion of young people with type 2 diabetes included within the study was smaller (>20%) than those with T1DM; there were variations in the age of transition (hence a broad range was adopted to accommodate this variation); and there have been a number of previous transition-related systematic reviews, although these reviews focussed on outcomes rather than the underpinning theory or delivery mechanisms of the interventions, which is the focus of this review.

### 2.1. Search Strategy

An electronic search of the following databases: Medline, Embase, HMIC (via OvidSP platform), CINAHL, and Child Development and Adolescent Studies (via EBSCO platform). Major international randomised and non-randomised clinical trial registries such as ISRCTN, ClinicalTrials.gov and ANZCTR were also searched. The websites of relevant professional and diabetes specialist bodies, including Diabetes UK, the American Diabetes Association, the Royal College of Nursing, the American Academy of Paediatrics and the Royal College of Paediatrics for unpublished, ongoing work and conference proceedings were also included. The Got Transition US National Resource Centre and Healthcare Transition Research Consortium lists of transition research were included. Hand searches of the included studies' reference lists were conducted. The search was last updated on January 5th, 2024.

The searches were constructed following key terms, formulated from a facet analysis informed by the scoping search as follows:

 
*Population 1* (*diabetes*): T1DM, Juvenile diabetes, insulin-dependent diabetes, diabetic, T1 diabetes, T1DM, Type1 diabetes.
 
*Population 2* (*adolescence*): Adolescent, pubescent, teen, paediatric, child, young people, young person, young adult, youth, juvenile, young man, young women, young male, young female.
 
*Intervention*: Transition, health transition, healthcare transition, patient transition, transition to adult care, transfer, patient transfer, care change, care continuity, care planning, HCT.

The search strings incorporated index and free text terms, combined using Boolean operators. Given the review's purpose and the variable nature of the outcomes evaluated, terms for the comparator and outcome domains were not specified. A learning technologist with expertise in optimising search strategies was consulted in developing the searches employed for the review (the full search strategies for each database are available in *Supporting Information 1*.

### 2.2. Inclusion and Exclusion Criteria

When assessing studies for inclusion, explicit selection criteria were established before the screening process to minimise the potential of selection bias and enable a repeatable, transparent search process. The inclusion criteria were based on the review aim and questions and aligned with the search terms adapted from the population intervention comparison outcomes framework components, without comparisons or outcomes, as follows [16]:

*Participants*: Young people with T1DM aged 10–25 years old.

*Interventions*: Any intervention that explicitly addressed the transition/transfer from paediatric to adult healthcare.

Only interventional studies were considered. The review was not confined to a specific healthcare setting or context. Studies were only included if they were reported in English; however, language filters were not applied during the search process.

*Limitations*: Only research published after 2000 was considered, since much has changed and progressed in the diabetes care context in recent decades [18].

The review's protocol was registered prospectively with the International Prospective Register of Systematic Reviews (PROSPERO) under the number CRD42021290893.

### 2.3. Screening

The citations from the search results were imported into Covidence to facilitate the screening process and to automatically eliminate duplicates. Titles and abstracts were screened by the lead author (NA) against eligibility criteria, with any uncertainties resolved by discussion (AF, SD and MD). After that, studies were evaluated for full-text eligibility by all authors (NA, AF, SD and MD). When the full text was unavailable, attempts were made to contact the study's authors in addition to searching the authors' profiles on Google Scholar and ResearchGate. Because some studies had multiple publications, the most recent record was considered. The titles of the studies cited in the included studies were then hand-searched to identify studies that the electronic search might have missed. The final decision to include studies was based on the explicit inclusion and exclusion criteria by (NA) in discussion with all authors.

### 2.4. Data Extraction

Data extraction of key information from the included studies was undertaken using a standardised electronic template that was developed and imported to Covidence Extraction 2.0. [19]. The template included data extraction fields relevant to the study objectives and incorporated the Cochrane Collaboration Form for data extraction for RCTs and non-RCTs [20] and included JBI data extraction criteria relating to study design and participants [16]. The extraction template also incorporated elements related to the MRC complex intervention framework: intervention components, underpinning theory and delivery mechanisms [17]. The template was piloted on four studies and refined.

### 2.5. Critical Appraisal of Studies

The included studies were critically appraised with the JBI check lists relevant to the study design employed. The appraisal was conducted by one author (NA) and then reviewed by three other authors (AF, SD and MD). Any disagreements were discussed until a consensus was reached. Experimental studies were appraised using the JBI Critical Appraisal Checklist for Randomised Controlled Trials (JBI-RCT); non-RCT studies were reviewed using the Quasi-Experimental (JBI-QE) tool. The JBI-RCT checklist is composed of 13 questions, while the JBI-QE has nine; these scores were used to rate the quality of each study as either high, moderate or low. Each question requires a ‘yes', ‘no' or ‘unclear' response; in some instances, a ‘not applicable (NA)' response where appropriate [16].

### 2.6. Data Synthesis

The extracted data were synthesised in a tabular form to provide an overview of the interventions' theories, components and delivery mechanisms together with the reported outcomes. As some studies did not provide explicit details of the underpinning theory or intervention components, it was necessary to interpret these from the descriptions provided where possible. A narrative synthesis was used to describe the main aspects of the studies under review, which considered converging and diverging approaches to healthcare transition. These approaches meant that the extracted findings could be presented in a uniform style, making clear the similarities and differences across the interventions analysed, and gave some scope for modelling the approaches that have been used in the narrative [21].

### 2.7. Search Outcomes

**Figure 1:**
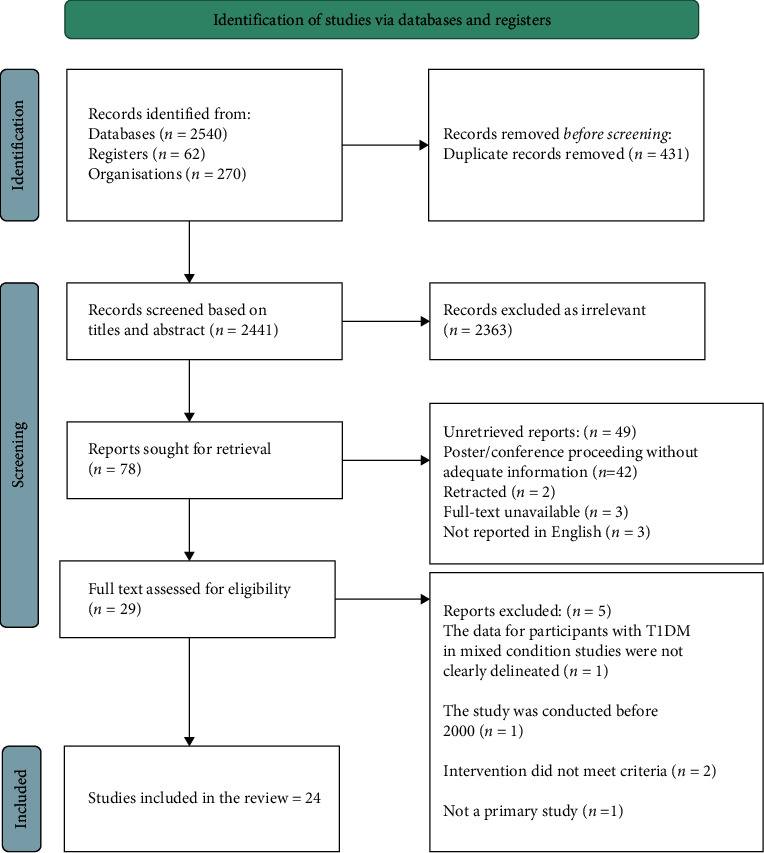
PRISMA chart.

The citations from the search results were imported into Covidence to facilitate the screening process and automatically eliminate duplicates. Titles and abstracts were screened by the lead author (NA) against eligibility criteria, with any uncertainties resolved by discussion with (AF, SD and MD). Accordingly, 2363 studies were excluded based on their potential relevance to the study criteria, leaving 29 studies that were reviewed in full. A further five records were eliminated for the reasons specified in Figure 1, with the remaining 24 eligible studies included. The Preferred Reporting Items for Systematic Reviews and Meta-analyses (PRISMA) flow diagram in Figure 1 summarises the results of the search and the selection criteria [22].

## 3. Results

### 3.1. Overview of Included Studies

The publication dates of the included studies ranged from 2006 to 2022; the median duration of these studies was 2 years. Six studies were conducted in the United States, five in Canada, three in Australia, eight in Europe (United Kingdom, Germany, Italy and Spain) and two in the Middle East (Israel and Tunisia). Many of the studies were observational, with six studies being retrospective clinical audits. Seven experimental studies were included, three of which were RCTs. T1DM-specific data could be clearly defined in three studies that included samples of young people from other chronic health conditions [23–25].  Table 1 shows a summary of the studies' characteristics and participants in addition to categories of transition intervention components and outcomes. Almost half of the studies included were rated poor in quality, with the other half being rated moderate quality.

### 3.2. Intervention Characteristics

The reviewed interventions were comprised of several components. These were grouped according to their objectives, and, as a result, the following seven different intervention component categories were identified, and these are described below:

1. Transition planning: This relates to the mechanisms used to prepare for the transition; they were identified in eight studies [29, 32, 35, 37, 38, 41]. These comprised both written and spoken formats. Written transition plans included either printed or digital materials (including medical records) explaining the anticipated transfer procedure, while verbal planning involved discussions either with young people and or their parents and/or between clinical teams in the adult and paediatric services [23, 24]. One study included the use of a series of questions to prepare the young person and their family to face the anticipated transitional challenges [44]. The level to which the young people were involved in the planning process varied. In one study, young people had input into their own transition medical records and discussed their plans with HCPs [35]. In other studies, the transition planning was more organisational, centred on communication between clinicians in the paediatric and adult services [35, 37]. In one study, the transition planning involved a transition readiness assessment [34]. There were variations in the point at which transition planning occurred. Early transition planning with young people, as a method to help them develop more autonomy in their diabetes care, was employed in some interventions [15, 43].
2. Service coordination: Service coordination was the most common component identified in transition interventions, being reported in 18 studies [6, 8, 23–25, 27–37, 39, 42]. Some interventions included processes aimed at coordinating the transition experience for young people and their families; and/or to integrate adult and paediatric services [43]. This category of interventions included: orientating young people to adult appointment systems and management processes [36, 44]; a transition coordinator role [35]; or promoting continuity of care by rotating diabetes HCPs across paediatrics and adult services to increase familiarity and understanding between the young person and the HCP pre- and post-transition [28]. Three interventions included the use of newsletters, websites and social media targeted at young people to promote awareness of the transition to adult care and how to navigate it [29, 35, 40]. The social media networks used were either private or generic; for instance, a designated Instagram account for the transition programme [40].
3. Transition clinics: The study interventions described several models of transition clinics. The clinic's design varied in relation to its scope, staffing and scheduling. Most of the transition clinic designs were modelled to the needs of the local settings and existing service configurations. Some models included an age-specific young adult transition clinic [23, 30, 35]. Again, there were variations in how these were operationalised; some were ongoing throughout the transition process [34, 43], although the majority were one-off joint clinics [27, 33, 39]. These appointments were generally conducted at the point of transition rather than over a period to prepare for the transition. Another element of the identified transition clinics was youth-oriented scheduling, with evening and weekend appointments [26, 40].
4. Pre-transition education: The provision of education to help young people prepare for transition was an element in many of the interventions. Ten studies reported interventions that comprised some form of education. The most common model of education offered was group-based and designed to provide peer support [27, 30, 31, 40]. In one study, the education was delivered via a printed information pack [39]. The education was not exclusively about the transition itself but incorporated elements to help prepare young people for greater autonomy in managing their condition; these mainly focussed on developing self-management skills, such as insulin adjustment and carbohydrate counting [30, 35, 37]. The details of the education programmes were not clear in several studies [30, 35], and none had a theoretical model underpinning the education. Hence, it was difficult to establish what the different mechanisms or active ingredients were in these interventions and how they might impact on the young person's transition experience.
5. Prompting and follow-up strategies: Communication strategies to boost the attendance of young people at adult appointments following transition were included in five of the identified interventions [23, 27, 34, 39, 40]. The communication processes included text messages, phone calls, helpline, email or a combination of these to contact young people regarding their healthcare transition. The main purpose of the communication was to simply remind young people of their initial adult care appointments [23, 34, 39] or to offer them support outside of the service's regular hours [40]; none involved motivational or supportive messaging to enhance the confidence of the young person in attending.
6. Paediatric and adult HCPs communication: Six of the interventions detailed communication strategies to enhance the connectivity between paediatric and adult care providers [8, 29, 34, 37, 38, 40]. These communication strategies were employed in some instances to support continuity, as the adult healthcare service was separately managed or located within a different setting from the paediatric service. Other communication components included discharge letters, referral letters and a diabetes summary passport detailing the clinical and care context of each young person [23, 25, 29, 31, 43]. In one intervention where the adult and paediatric teams were co-located, communication involved regular multidisciplinary case discussion meetings during the transition period [29].

Parent engagement: Very few interventions were explicitly targeted at or involved parents; only five studies included it as a component [23, 29, 32, 38, 44], and the level of parental involvement varied greatly across these studies. In the Clover et al. [23] study, the involvement of parents was passive, whereas the Mistry et al. [32] intervention actively included parents when giving instructions on how to navigate adult services. In the Cadario et al. [29] and Essaddam et al. [38] studies, parents were invited to attend joint paediatric and adult appointments and discussions with the young person.

The profile of component categories across the studies is presented in Table 2.

### 3.3. Underpinning Theory

Surprisingly, only two of the studies identified explicitly used a theoretical framework to guide the implementation of their intervention strategies [25, 27–38, 41, 43, 44]. Agarwal et al. [36] employ the social–ecological model of adolescent and young adult readiness to transition (SMART) framework, which is a social–behavioural theory. The SMART approach involves identifying modifiable and unmodifiable elements that influence the likelihood of a successful transition to adult-oriented care. SMART was developed to respond to the complex, dynamic, and social–ecological context of the healthcare transition. The framework was created, considering existing literature, undergoing expert review and conducting pilot data collection as part of a childhood cancer survivorship programme [45]. Schmidt et al. [25] used Zimmerman's empowerment theory as a framework for their intervention. This theory conceptualises empowerment as having an intrapersonal component (the capacity to affect a situation), an interactional component (knowing how the system operates) and a behavioural component (engaging in behaviours exerting control) [46].

Of the two theoretical models identified, the SMART model was more comprehensive as its focus extends beyond the young person's knowledge and skills about their condition to include inter-relational dynamics with parents and health professionals and preparing young people to become more autonomous in relation to their condition. It also considers transition readiness and the psychosocial well-being of young people, their parents and their healthcare providers [45]. In terms of limitations, SMART does not consider the complexity of individual experiences. Furthermore, it is not clear whether the model's cancer-specific aspects could be transferred to the diabetes care context. In contrast, Zimmerman's theory is exclusive to empowering young people to develop their personal autonomy without recognising the inter-relational dynamics, socio-cultural context or limitations in a healthcare organisation [46]. It was unclear in both studies how the theories were integrated with the different intervention components; hence, it was difficult to explicate the relationship between both frameworks and the mechanism of the interventions.

### 3.4. Intervention Delivery

The interventions varied in relation to their duration and timing, and who was involved in delivering the intervention. The different facets of intervention delivery are outlined below:

1. Intervention duration: A third of the included studies did not report the duration of their interventions. When duration was reported, it was difficult to distinguish between the length of study participation and the duration of the intervention itself. The average length of time spent in transition, where reported in the studies, was 12 months [30, 31, 33], although the duration of exposure to individual components was not explicit and many of these were episodic in nature, such as attending a group session or general communication between adult and paediatric services.
2. Transition timing: Transition timing varied greatly between studies. Most studies indicated that the age of 18 years was the timepoint of transition and the focus of the intervention, while the youngest transition age reported was 14 years [6]. Some studies adopted a more individualised approach, referring to transition ‘readiness', on the part of the young person, as the criterion for determining transition timing [26, 34]. In one study from Australia, this involved the use of a standardised checklist to assess for transition readiness [34].
3. Intervention providers: Most of the interventions were delivered by multidisciplinary teams of healthcare professionals. A few interventions, however, were delivered exclusively by physicians [37, 41]. In the transition model adopted by Spaic et al. [8], a specific transition role was identified; in this case, it was a physician who was trained in both adult and paediatric endocrinology. Other studies employed a non-medical professional to support the transition, included a designated patient navigator or coordinator, administrative assistants or secretaries [6, 30, 36].
4. Intervention recipients: Most of the interventions were exclusively targeted at young people, with only three studies also incorporating parental involvement [29, 30, 40]. In two studies, health-care professionals were also identified as intervention recipients through participating in transition planning meetings or case discussions [8, 40].
5. Intervention resources: The explicit resources required to support intervention delivery included access to an appropriate venue, clinical and HCP time, development of communication systems, the use of electronic health records[43] and providing training resources.

### 3.5. Reported Outcomes

The included studies used a wide range of patient-level outcome measures; these were grouped into five different outcome categories: metabolic outcomes, diabetes complications, care engagement, self-management behaviours and psychological impact. A full summary of the outcome categories and the distribution of outcomes across studies is presented in Table 3 (full details of study outcomes can be found in *Supporting Infromation 2*). There were inconsistencies between the studies in relation to how each outcome was defined, measured or reported. Consequently, determining the average effects on outcomes across studies was not possible, although Table 3 indicates what was and what was not measured across the studies and whether they reported positive outcomes or not. The majority of the reported outcomes were metabolic (HbA1c) or related to acute diabetes complications (DKA or severe hypoglycaemia). The reporting of impact on psychosocial or self-management outcomes was limited; the measures used were heterogeneous and included diabetes or general quality of life (QoL), diabetes empowerment, self-efficacy and frequency of blood glucose monitoring. The improvement or worsening of metabolic outcomes shown in the table was based on statistical significance (*p* ≤ 0.05); for HbA1c, the change had to be both statistically and clinically significant (defined as a reduction ≥0.05%).

**Figure 2:**
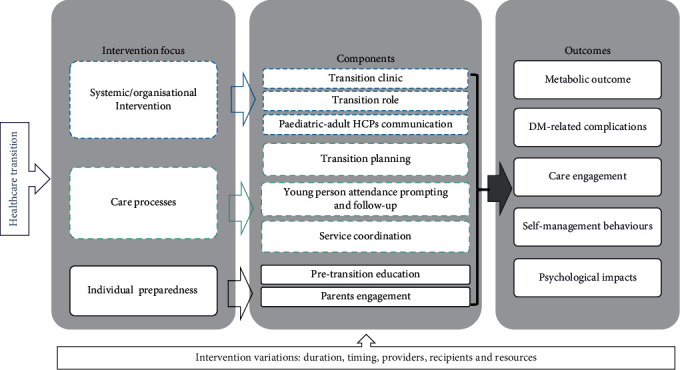
A logic model that depicts the shared relationships among the resources, activities, components, outputs, outcomes and impact of the transition interventions reviewed.

Figure 2 illustrates a logic model that depicts the shared relationships among the resources, activities, components, outputs, outcomes and impact of the transition interventions reviewed.

## 4. Discussion

This review has systematically identified and synthesised existing evidence relating to transition interventions in adolescents with T1DM. Unlike previous reviews of transition interventions topic [13–15], this review has focussed on the nature of the interventions in relation to underpinning theory, active components and delivery methods. This review has highlighted that a wide range of intervention strategies involving young people, parents and health professionals have been reported. Most of the interventions were multimodal, reflecting the complex nature of the transition. The different intervention components targeted: systemic/organisational changes (integrated clinics, transition roles and HCP communication); care processes (transition planning, prompts and reminders to activate care attendance during the transition); and personal preparation (transition education, information via different media, developing self-management autonomy and peer support). Based on the presented outcome data, it is difficult to identify the overall impact of these interventions due to the large variability in what was measured and in the reported effects. One of the key areas of weakness within the reviewed studies was a lack of a theoretical perspective on the transition and in relation to detailing the theoretical underpinnings of the studies.

Overall, the interventions tended to emphasise the transfer itself (moving from paediatric to adult care); rather than the transition process, which addresses the biopsychosocial developmental changes that are occurring in this period [2]. It has been suggested by previous reviews that transitional care needs to be phasic following a structured transitional care programme that addresses the developmental needs of the young person to allow them to build personal autonomy in relation to their diabetes [9]. It has been proposed that successful transition management for young people with T1DM requires a sequential model that combines interventions such as joint adult and paediatric working and parental involvement within a developmental framework. This framework considers the readiness of the young person for transition and fosters their autonomy so they can navigate and effectively engage with adult diabetes care [12]. In most of the reviewed interventions, the readiness and timing were based on chronological years and did not accommodate variability in the personal development of the young person or the impact of cultural contexts on the readiness to transition [47].

While two of the studies included theoretical models that addressed the developmental elements of the transition process, it was not clear how these theories were enacted in the described interventions [25, 36]. These studies were among the few to report positive impacts on self-management. The Schmidt et al. [25] study also reported a positive impact on self-efficacy and transition competence. However, overall, the reported studies lacked or failed to detail any underpinning theory, limiting the potential transferability of these interventions [17]. The evidence from the studies conducted to date suggests that optimal transition care conceptual models for young people with T1DM are yet to be fully identified and tend to focus on transfer rather than transition [4, 48].

The transition components reviewed were variable in both their focus and delivery. Where some of the interventions incorporated multiple components as recommended by the Healthcare Transition Consortium transition [4, 48], others were only comprised of one or two. It was not possible to assess from the multimodal interventions the impact of individual components on study outcomes. As illustrated in Table 3, most of the interventions centred on the young person service focus continuum rather than transition as a developmental process [12]. While service organisation is an important aspect of the healthcare transition, developmental domains such as individual, family and social support, as well as the environment, will influence the success of adolescents' integration into adult-focused healthcare services [49]. These domains are largely unaddressed in the studies reviewed.

In terms of reported outcomes, the review provides a comprehensive overview of the variation in outcomes and of the tools used to measure outcomes across the included studies. The inconsistent definition, measurement, and reporting of outcomes pose a challenge to determining the average effects across studies. This lack of standardisation creates difficulties in comparing results and drawing meaningful conclusions [50]. Similar to the objective of the transition components identified, the focus of the outcomes was predominantly on service coordination and metabolic outcomes. However, while biophysiological outcomes are a crucial aspect of the transition process, the review highlights a lack of emphasis on the psychosocial context of the transition. This is concerning as the psychosocial and self-management outcomes provide valuable insight into the broader impact of the transition on the individuals undergoing it and will have a significant determining effect on the long-term health of young people with diabetes.

Therefore, it is important in designing future interventions that they should be theoretically modelled with explicit details of the active ingredients and mechanisms within the intervention and how these relate to the reported outcomes. It would also be important for future interventions to consider the process of transition rather than the transition itself, recognising the needs of the young person and their parents as well as the service context and structure. Furthermore, to overcome the challenge of determining average effects across studies, future research should strive for standardisation in the definition, measurement and reporting of outcomes [50]. Some convergence in outcomes would help ensure that results are comparable. The findings of this review also suggest that future studies should broaden the scope of outcomes and place a greater emphasis on psychosocial outcomes as well as care engagement, activation and adaption to provide a more comprehensive understanding of the transition process and its impact on young people, their parents, and HCPs.

## 5. Strengths and Limitations

While the review adhered to a recommended review framework and employed a comprehensive search strategy, there were some limitations to the findings related to the quality, design, focus and context of the available studies. First, studies in this review were mainly conducted in the United States, the United Kingdom, Canada and Australia, with a noticeable absence of studies from low-to-middle-income countries. Studies from the United States, in particular, were often contextually different to studies from other countries due to the nature of their health insurance-based care system [51]. Second, given the generally poor quality of the primary research studies, the lack of intervention details, and the wide variation in outcome measures used, it was not possible to determine which components were key to successful transitional interventions in young people with T1DM.

## 6. Conclusion

In conclusion, the review findings indicate that transition interventions have primarily focused on a service-oriented context, with a limited consideration of the personal development and complexities that occur in the adolescence phase of life during the transition period. Furthermore, many of the studies reviewed tend to emphasise transfer as a single event rather than the process of transition, which is much broader and involves developing self-identity and personal autonomy, taking more responsibility for their personal health, and changes in interpersonal relations, including with parents and HCPs. The review emphasises the need for theory-based and modelled interventions that address the transition from multiple perspectives; and for a core outcome set for assessing impact and effectiveness.

## Figures and Tables

**Table 1:** Summary of evidence.

Study citation, year, country, design	Aim	Participants		Transition intervention category	Outcome category	Quality assessment
		Total number	Age (years)			
1- Johnston et al. [26], the United Kingdom, Clinical audit	To evaluate attendance before and after transfer and identify reasons for non-attendance.	37	Range: 14–24	Transition clinic	Metabolic control:(HbA1c) Care engagement: clinic attendance	Low

2- Lane et al. [27], the United States, Clinical audit	To assess the impact of a specialised young adult diabetes clinic on HbA1c in young adults with T1DM transitioning from paediatric to adult diabetes care.	249	Mean ± SD: 19 ± 2	Transition clinic	Metabolic control: (HbA1c)	Low

3- Nakhla et al. [28], Canada, Cohort study	- To compare pre- and post-transition rates of DM-related hospitalisation and retinopathy screening - To assess if different methods of care transfer led to better outcomes.	1507	Range: 18–20	Other: change of DM team and/or physician (transfer)	DM-related complications: DM-related hospitalisation Care engagement: Retinopathy screening visits	Moderate

4- Cadario et al. [29], Italy, Clinical audit	To compare the outcomes of a structured transition versus unstructured transition in a population with T1DM.	62	Mean ± SD: 19.0 ± 2.8	Transition programme	Metabolic control: (HbA1c) Care engagement: clinic attendance	Low

5- Van Walleghem et al. [30], Canada, Cohort study	To assess the acceptability and effectiveness of a non-health professional patient navigator (the Maestro Project) on medical outcomes.	165	Young group: 18 Older group Range: 19–25	Transition role	Care engagement: Clinic attendance DM-related complications: DKA episodes Severe hypoglycaemia episodes Pregnancy loss, Legal blindness, Heart failure Amputation Death	Low

6- Helgeson et al. [31], the United States, Cohort study	To describe the transition of youth with T1DM from paediatric to adult care.	127	Median: 16.2	Transition programme	Metabolic control: (HbA1c) Self-management behaviours: self-care Psychosocial impacts: parent–child relationship	Low

7- Mistry et al. [32], Canada, Non-randomised experimental study	To assess young people's attendance to adult care after discharge from a structured Diabetes Transition Clinic.	136	NR	Transition clinic	Metabolic control: (HbA1c) Care engagement: eye examination attendance Rate of asking questions during the diabetes transition clinic appointment Self-management behaviours: self-reported frequency of home glucose testing	Moderate

8- Sequeira et al. [33], the United States, Non-randomised experimental study	To compare the efficacy of a structured transition programme to standard care.	81	Mean ± SD: 19.61 ± 1.02	Transition programme	Metabolic control: (HbA1c) DM-related complications: incidents of severe hypoglycaemia, Tertiary healthcare setting utilisation, Psychosocial impacts: diabetes empowerment Global wellbeing Depression Other: diabetes knowledge	Moderate

9- Steinbeck et al. [34], Australia, RCT	To compare a comprehensive transition programme with standard clinical practice for people with T1DM.	26	Mean ± SD: 17.3–18.8	Transition role	Metabolic control: (HbA1c) Care engagement: adult care retention DM-related complications: DM-related hospitalisations Microvascular complication incidence Psychosocial impacts: global self-worth	Moderate

10- Levy-Shraga et al. [35], Israel, Cohort study	To evaluate HbA1c, clinic attendance, acute complications, and quality of life (QoL) of people attending a transition clinic.	53	Mean ± SD: 22.1 ± 2.7	Transition clinic	Metabolic control: (HbA1c) DM-related complications: DKA and severe hypoglycaemia event Care engagement: clinic attendance Psychosocial impacts: diabetes-QoL.	Moderate

11- Schmidt et al. [25], Germany, Non-randomised experimental study	To assess the impact of a generic transition-oriented patient education programme on adolescent health-care participation and QoL.	325	Mean ± SD: 16.80 ± 1.74	Transition programme	Care engagement: young person activation Psychosocial impacts: QoL Self-efficacy Other: health-related transition competence and satisfaction with healthcare	Moderate

12- Agarwal et al. [36], the United States, Cohort	To assess an adult health care programme model for emerging adults with T1DM transitioning from paediatric to adult care.	122	Mean ± SD: 20.2 ± 1.7	Transition clinic	Metabolic control: (HbA1c) Care engagement: clinic attendance Self-management behaviours: blood glucose monitoring frequency	Moderate

13- Jones et al. [24], the United States, Clinical audit	To establish a systematic health care transition (HCT) process in subspecialty practises to increase successful transfer to adult care.	371	Mean ± SD: 21.15 ± 2.15	Transition programme	Metabolic control: (HbA1c) DM-related complications: tertiary care utilisation Care engagement: the period between last paediatric and first adult visits, The proportion of adolescents referred to and attending the adult endocrinology practice	Low

14- White, O'Connell and Cameron [37], Australia, RCT	To evaluate the impact of an appointment-management intervention on clinic attendance and disengagement following the transition.	120	Mean ± SD: 18·8 ± 0·6	Transition role	Metabolic Control: (HbA1c) Care engagement: clinic attendance Disengagement from services Frequency and timing of participant contact by the appointment manager	Moderate

15- Essaddam et al. [38], Tunisia, Cohort study	To assess a novel North African structured transition programme consisting of transitional meetings involving both paediatric and adult teams attended by a young person.	65	Mean ± SD: 14.5–23.2	Transition programme	Metabolic control: (HbA1c) DM-related complications: rate of hospitalisation Care engagement: the gap between the last paediatric and first adult clinic visit Clinic attendance Other: changes to the treatment plan	Moderate

16- Farrell et al. [39], Australia, Clinical audit	To assess the impact of transitioning to a youth-specific diabetes service on young people with T1DM.	439	Median: 18	Transition programme	Metabolic control: (HbA1c) DM-related complications: Acute admissions with DKA	Moderate

17- Colver et al. [23], the United Kingdom, Cohort study	To explore the effect of a transition program on outcomes for young individuals with long-term illnesses with specific proposed beneficial features.	150	Mean ± SD: 16.2 ± 1.3	Transition programme	Care engagement: Participation and autonomy in appointments Psychosocial impacts: mental wellbeing Other: satisfaction with services	Moderate

18- Dalton et al. [40], the United Kingdom, Report	To explain how the service was established, how it operates today, and how it plans to evolve in the future.	NR	NR	Other: service (team)	Metabolic control: (HbA1c) DM-related complications: DM-related hospitalisation Care engagement: clinic non-attendance	Low

19- Lal et al. [41], the United States, Cohort study	To assess the role of a physician who has received training in both adult and paediatric endocrinology.	26	Mean ± SD: 20.3 ± 2.4	Transition role	Metabolic control: (HbA1c) DM-related complications: DKA hospitalisation Care engagement: adult clinic visits	Low

20- Spaic et al. [8], Canada, RCT	To evaluate a structured transition programme for young adults with T1DM on clinic attendance, glycaemic control, diabetes-related distress, QoL, and satisfaction with care.	205	Mean ± SD: 17.9 ± 0.7	Transition role	Metabolic control: (HbA1c) DM-related complications: diabetes-related ER visits Hospitalisations for DKA and hypoglycaemia Care engagement: clinic visits, Frequency of complications screening Psychosocial impact: diabetes distress Diabetes impact on quality of life Other: satisfaction with the transition process	Moderate

21- Flor et al. [42], Spain, Cohort study	To assess the outcomes of a healthcare and a therapeutic education programme aimed at young people with T1DM who had been transferred from a paediatric centre.	330	Mean ± SD: 18.2 ± 0.7	Transition programme	Metabolic control: (HbA1c) DM-related complications: frequency of mild/severe Hypoglycaemia Lipodystrophy Self-management behaviours: units of insulin Number of capillary blood glucose readings Psychosocial impacts: diabetes quality of life (DQoL) QoL in general Eating behaviours Others: diabetes knowledge Perception of hypoglycaemia symptoms	Low

22- Butalia et al. [6], Canada, Non-randomised experimental study	To compare the effectiveness of a communication technology-enhanced transition coordinator intervention to standard care.	202	Mean ± SD: 18 ± 0.4	Transition role	Metabolic control: (HbA1c) DM-related complications: DM-related emergency department (ED) visits DKA hospitalisations albumin/creatinine ratio Care engagement: clinic non-attendance Self-management behaviours: self-efficacy Psychosocial impacts: DQoL	Moderate

23- Pasquini et al. [43]	To assess the impact of a structured and joint paediatric-adult teams' meetings with psychological support on transitioning young people.	222	Mean ± SD: 24.4 ± 5.8	Transition Clinic	Metabolic control: (HbA1c) DM-related complications: diabetic retinopathy Diabetic nephropathy Psychosocial impacts: Disorders of eating behaviours Depressive and/or anxiety Fear of hypoglycaemia Diabetes acceptance	Moderate

24- Harmer et al. [44]	To assess the effectiveness of a structured transition tool	106	Median: 18 years	Transition planning	Metabolic control: (HbA1c) DM-related complications: DM-related hospital admissions Non-HDL cholesterol	Moderate

**Table 2:** Components of the transition interventions.

Study	Transition planning	Service coordination and navigation	Pre-transition diabetes education	Young person attendance prompting and follow-up	Online networks	Transition clinic	Youth-oriented scheduling	Parents' engagement	Multidisciplinary transition care	A joint paediatric and adult care appointment	Paediatric and adult HCPs communication
1	—	—	—	—	—	✓	✓	—	—	—	—
2	—	✓	✓	✓	—	✓	—	—	—	✓	—
3	—	✓	—	—	—	—	—	—	✓	—	—
4	✓	✓	—	—	—	—	—	✓	—	—	✓
5	—	✓	✓	—	✓	—	✓	—	—	—	—
6	—	✓	✓	—	—	—	—	—	—	—	—
7	✓	✓	—	✓	—	✓	—	✓	✓	—	—
8	—	✓	✓	—	✓	✓	—	—	—	✓	—
9	—	✓	—	✓	—	—	—	—	—	—	✓
10	✓	✓	—	—	—	✓	✓	—	✓	—	—
11	—	✓	✓	—	—	—	—	—	—	—	—
12	—	✓	✓	—	—	—	—	—	—	—	—
13	—	✓	—	—	—	—	—	—	—	—	—
14	✓	✓	✓	✓	—	—	—	—	—	—	✓
15	✓	—	—	—	—	—	✓	✓	—	—	✓
16	—	✓	—	—	—	✓	—	—	—	✓	—
17	—	✓	—	—	—	✓	—	✓	✓	—	—
18	—	—	✓	✓	✓	—	✓	—	✓	—	✓
19	✓	—	—	—	—	—	—	—	—	—	—
20	—	✓	✓	—	—	—	—	—	—	—	✓
21	—	✓	✓	—	—	—	—	—	✓	—	—
22	—	✓	—	—	—	—	—	—	✓	—	—
23	✓	✓	—	—	—	✓	—	—	✓	✓	✓
24	✓	✓	—	—	—	—	—	✓	✓	—	✓

**Table 3:** Changes in the measured outcomes.

Study	Metabolic Outcomes	DM-related complications	Care engagement	Self-management behaviours	Psychological impacts
1	+	NM	+	NM	NM
2	+	NM	NM	NM	NM
3	NM	−	−	NM	NM
4	+	NM	+	NM	NM
5	NM	−	+	NM	NM
6	+	NM	NM	+	+
7	NR	NR	NB	NB	NM
8	0	+	NM	NM	0
9	−	−	0	NM	NM
10	+	NB	NB	NM	+
11	NM	NM	NM	+	+
12	0	NM	NM	+	NM
13	0	0	+	NM	NM
14	−	−	0	NM	NM
15	0	+	NM	NM	NM
16	NM	NM	+	NM	0
17	NB	+	−	NM	NM
18	+	−	−	NM	NM
19	0	0	+	NM	0
20	0	0	+	NM	0
21	0	+	NM	NR	0
22	+	−	+	NR	NR
23	−	−	NM	NM	−
24	+	0	NM	NM	NM

*Note*: + Improved, 0 No effect, − Worsen.

Abbreviations: NB, no baseline; NM, not measured; NR, not reported.

## Data Availability

The data supporting this systematic review are from previously reported studies and datasets, which have been cited. The processed data are available in the supplementary files.

## Supporting Information

Supporting Information 1 Search strategy.

Supporting Information 2 Outcomes details.
