# Supplementary material for: The Journey to Adulthood: A Systematic Review of Interventions in Type 1 Diabetes Paediatric to Adult Transition Care
Source: Pediatr Diabetes. 2024 Sep 26;2024:1773726. doi: 10.1155/2024/1773726 (PMC12016725; doi:10.1155/2024/1773726)
Supplement: Supporting Information 1 — Search strategy. [file 1773726.f1.docx]

**Embase** <1974 to 2024 January 05>

Ovid **MEDLINE(R)** ALL <1946 to January 05, 2024>

**HMIC Health Management Information Consortium** <1979 to September 2023>

1 type1diabetes.mp. [mp=ti, ab, hw, tn, ot, dm, mf, dv, kf, fx, dq, bt, nm, ox, px, rx, ui, sy, ux, mx] 39

2 (type 1 adj diabetes).mp. [mp=ti, ab, hw, tn, ot, dm, mf, dv, kf, fx, dq, bt, nm, ox, px, rx, ui, sy, ux, mx] 138959

3 T1DM.mp. [mp=ti, ab, hw, tn, ot, dm, mf, dv, kf, fx, dq, bt, nm, ox, px, rx, ui, sy, ux, mx] 19856

4 (T1 adj diabetes).mp. [mp=ti, ab, hw, tn, ot, dm, mf, dv, kf, fx, dq, bt, nm, ox, px, rx, ui, sy, ux, mx] 138

5 (Insulin adj dependent diabetes).mp. [mp=ti, ab, hw, tn, ot, dm, mf, dv, kf, fx, dq, bt, nm, ox, px, rx, ui, sy, ux, mx] 483950

6 (Juvenile adj diabetes).mp. [mp=ti, ab, hw, tn, ot, dm, mf, dv, kf, fx, dq, bt, nm, ox, px, rx, ui, sy, ux, mx] 4724

7 1 or 2 or 3 or 4 or 5 or 6 551228

8 Pubescen*.mp. [mp=ti, ab, hw, tn, ot, dm, mf, dv, kf, fx, dq, bt, nm, ox, px, rx, ui, sy, ux, mx] 6641

9 Adolescen*.mp. [mp=ti, ab, hw, tn, ot, dm, mf, dv, kf, fx, dq, bt, nm, ox, px, rx, ui, sy, ux, mx] 4260433

10 teen*.mp. [mp=ti, ab, hw, tn, ot, dm, mf, dv, kf, fx, dq, bt, nm, ox, px, rx, ui, sy, ux, mx] 89248

11 child*.mp. [mp=ti, ab, hw, tn, ot, dm, mf, dv, kf, fx, dq, bt, nm, ox, px, rx, ui, sy, ux, mx] 6083673

12 P?ediatric.mp. [mp=ti, ab, hw, tn, ot, dm, mf, dv, kf, fx, dq, bt, nm, ox, px, rx, ui, sy, ux, mx] 1204064

13 (m?n or wom?n or person or people or male or female).mp. [mp=ti, ab, hw, tn, ot, dm, mf, dv, kf, fx, dq, bt, nm, ox, px, rx, ui, sy, ux, mx] 31270257

14 young.mp. [mp=ti, ab, hw, tn, ot, dm, mf, dv, kf, fx, dq, bt, nm, ox, px, rx, ui, sy, ux, mx] 2744248

15 13 and 14 2287278

16 Juvenile.mp. [mp=ti, ab, hw, tn, ot, dm, mf, dv, kf, fx, dq, bt, nm, ox, px, rx, ui, sy, ux, mx] 271018

17 youth.mp. [mp=ti, ab, hw, tn, ot, dm, mf, dv, kf, fx, dq, bt, nm, ox, px, rx, ui, sy, ux, mx] 218060

18 8 or 9 or 10 or 11 or 12 or 16 or 17 8501045

19 (transition* or transfer* or change or continuity or planning).mp. [mp=ti, ab, hw, tn, ot, dm, mf, dv, kf, fx, dq, bt, nm, ox, px, rx, ui, sy, ux, mx] 7358205

20 ((care or health) adj care).mp. [mp=ti, ab, hw, tn, ot, dm, mf, dv, kf, fx, dq, bt, nm, ox, px, rx, ui, sy, ux, mx] 2971084

21 19 and 20 507560

22 HCT.mp. [mp=ti, ab, hw, tn, ot, dm, mf, dv, kf, fx, dq, bt, nm, ox, px, rx, ui, sy, ux, mx] 68398

23 21 or 22 575462

24 7 and 18 and 23 1551

25 limit 24 to yr="2022 - 2024" 243

26 type1diabetes.mp. [mp=ti, ab, hw, tn, ot, dm, mf, dv, kf, fx, dq, bt, nm, ox, px, rx, ui, sy, ux, mx] 39

27 (type 1 adj diabetes).mp. [mp=ti, ab, hw, tn, ot, dm, mf, dv, kf, fx, dq, bt, nm, ox, px, rx, ui, sy, ux, mx] 138959

28 T1DM.mp. [mp=ti, ab, hw, tn, ot, dm, mf, dv, kf, fx, dq, bt, nm, ox, px, rx, ui, sy, ux, mx] 19856

29 (T1 adj diabetes).mp. [mp=ti, ab, hw, tn, ot, dm, mf, dv, kf, fx, dq, bt, nm, ox, px, rx, ui, sy, ux, mx] 138

30 (Insulin adj dependent diabetes).mp. [mp=ti, ab, hw, tn, ot, dm, mf, dv, kf, fx, dq, bt, nm, ox, px, rx, ui, sy, ux, mx] 483950

31 (Juvenile adj diabetes).mp. [mp=ti, ab, hw, tn, ot, dm, mf, dv, kf, fx, dq, bt, nm, ox, px, rx, ui, sy, ux, mx] 4724

32 26 or 27 or 28 or 29 or 30 or 31 551228

33 Pubescen*.mp. [mp=ti, ab, hw, tn, ot, dm, mf, dv, kf, fx, dq, bt, nm, ox, px, rx, ui, sy, ux, mx] 6641

34 Adolescen*.mp. [mp=ti, ab, hw, tn, ot, dm, mf, dv, kf, fx, dq, bt, nm, ox, px, rx, ui, sy, ux, mx] 4260433

35 teen*.mp. [mp=ti, ab, hw, tn, ot, dm, mf, dv, kf, fx, dq, bt, nm, ox, px, rx, ui, sy, ux, mx] 89248

36 child*.mp. [mp=ti, ab, hw, tn, ot, dm, mf, dv, kf, fx, dq, bt, nm, ox, px, rx, ui, sy, ux, mx] 6083673

37 P?ediatric.mp. [mp=ti, ab, hw, tn, ot, dm, mf, dv, kf, fx, dq, bt, nm, ox, px, rx, ui, sy, ux, mx] 1204064

38 (m?n or wom?n or person or people or male or female).mp. [mp=ti, ab, hw, tn, ot, dm, mf, dv, kf, fx, dq, bt, nm, ox, px, rx, ui, sy, ux, mx] 31270257

39 young.mp. [mp=ti, ab, hw, tn, ot, dm, mf, dv, kf, fx, dq, bt, nm, ox, px, rx, ui, sy, ux, mx] 2744248

40 38 and 39 2287278

41 Juvenile.mp. [mp=ti, ab, hw, tn, ot, dm, mf, dv, kf, fx, dq, bt, nm, ox, px, rx, ui, sy, ux, mx] 271018

42 youth.mp. [mp=ti, ab, hw, tn, ot, dm, mf, dv, kf, fx, dq, bt, nm, ox, px, rx, ui, sy, ux, mx] 218060

43 33 or 34 or 35 or 36 or 37 or 41 or 42 8501045

44 (transition* or transfer* or change or continuity or planning).mp. [mp=ti, ab, hw, tn, ot, dm, mf, dv, kf, fx, dq, bt, nm, ox, px, rx, ui, sy, ux, mx] 7358205

45 ((care or health) adj care).mp. [mp=ti, ab, hw, tn, ot, dm, mf, dv, kf, fx, dq, bt, nm, ox, px, rx, ui, sy, ux, mx] 2971084

46 44 and 45 507560

47 HCT.mp. [mp=ti, ab, hw, tn, ot, dm, mf, dv, kf, fx, dq, bt, nm, ox, px, rx, ui, sy, ux, mx] 68398

48 46 or 47 575462

49 32 and 43 and 48 1551

**CINAHL and Child Development & Adolescent Studies via EBSCO**

**January 09, 2024**

( type 1 diabetes mellitus OR T1DM OR t1 ADJ diabetes OR Juvenile ADJ diabetes OR insulin-dependent ADJ diabetes OR type 1 ADJ diabetic OR type1diabetes ) AND ( adolescen* OR pubescen* OR teen* OR young ADJ adult OR child* OR p?ediatri* OR young ADJ wom#n OR young ADJ m#n OR young ADJ female* OR young ADJ male* OR juvenile OR youth ) AND ( transition* OR health ADJ transition* OR healthcare ADJ transition* OR HCT OR tranfer* OR care ADJ change OR care ADJ planning OR care ADJ continuity OR transition ADJ1 adult ADJ care OR patient ADJ transition OR patient ADJ tranfer* ) Limiters - Publication Date: 20000101-20211231

Expanders - Apply equivalent subjects

Search modes - Boolean/Phrase Interface - EBSCOhost Research Databases

Search Screen - Advanced Search

Database - CINAHL;Child Development & Adolescent Studies 327
