# Supplementary material for: The Journey to Adulthood: A Systematic Review of Interventions in Type 1 Diabetes Paediatric to Adult Transition Care
Source: Pediatr Diabetes. 2024 Sep 26;2024:1773726. doi: 10.1155/2024/1773726 (PMC12016725; doi:10.1155/2024/1773726)
Supplement: Supporting Information 2 — Outcomes details. [file 1773726.f2.docx]

**Full Details of Studies Outcomes**

**NM= not measured**

| Study | **Metabolic outcome (HbA1C)** | **Care engagement** | **DM-related complications** | **Self-Management behaviours** | **Psychosocial impact** |
| --- | --- | --- | --- | --- | --- |
| 1 | **Before transfer:**  <7.6% in 21%  7.6-9.0% in 24%  >9.0% in 55%  **After transfer:**  <7.6% in 37%  7.6-9.0% in 37%  >9.0% in 26% | **Clinic attendance**  **Before transfer:**  30% poor  18% moderate  52% good  **After transfer:**  30% poor  33% moderate  38% good | **NM** | **NM** | **NM** |
| 2 | **Baseline:**  9.0±2.3  **At 6 months:**  8.6±1.9  **At 12 months:**  8.7±2.1  **At 2 years:**  8.5±1.9  **At 3 years:**  8.6±2.1  (P=0.55) | **NM** | **NM** | **NM** | **NM** |
| 3 | **NM** | **Percentage of people attending more than one eye care examination.**  **Before transition:**  72%  **After transition:**  70% | **DM-related hospitalizations**  **Before transition:**  7.6 cases per100 patient-years  (P=.03)  **After transition:**  9.5 cases per100 patient-years. | **NM** | **NM** |
| 4 | **Control group:**  8.9±0.5 %  P<0.01  **Intervention group:**  8.9±0.5 %  P<0.01 | **Adult clinic attendance**  **Control group:**  57.0±5.0 %  P<0.05  **Intervention group:**  80.0± 12.5 % | **NM** | **NM** | **NM** |
| 5 | **NM** | **Attending more than one medical appointment**  **Control group:**  Before transition 59.4%  After transition 73.3%  **Intervention group:**  Before transition: 95.3%  After transition: 89.1%  **Attending more than one diabetes Educator appointment**  **Control group:**  Before transition: 25.7%  After transition: 41.6%  **Intervention group:**  Before transition:92.2%  After transition:53.1% | **Control group (older age):**  3.0 cases/100 patient-years of DKA  2.4 cases/100 patient-years of hypoglycaemia  38% pregnancy loss.  1 heart failure.  1 legal blindness.  1 amputation.  4 proliferative diabetic retinopathies.  4 deaths (2 DKA, 2 non-DM-related).  **Intervention group (younger age):**  7.9 cases/100 patient-years of DKA.  4.7 cases/100 patient-years of severe hypoglycaemia.  No long-term complications. | **NM** | **NM** |
| 6 | **Changes of glycaemia outcome over time (%)**  **Before transfer:**  Pediatric group (97%)  **After transfer:**  Adult group (36%)  Transition group (74%)  P<0.05 | **NM** | **NM** | **Changes of self-care over time (%)**  **Before transfer:**  Paediatric group (97%)  **After transfer:**  Adult group (92%)  Transition group (89%)  P<0.005 | **Parental involvement (mean)**  **Before transfer:**  Paediatric (3.66)  **After transfer:**  The transition group (2.82)  Adult group (3.24)  P=0.001 |
| 7 | **HbA1c (%) (average)**  **Before transfer:**  9.3%  **After transfer:**  **NR** | **Eye examination attendance**  **Before transfer:**  81.5%  **Asking question during appt**  **Before transfer:**  39.6%  **After transfer:**  **NR** | **NM** | **Frequency of home glucose monitoring (mean)**  **Before transfer:**  1.5±0.50 times per day  **After transfer:**  **NR** | **NM** |
| 8 | **Mean HbA1C**  **Before transfer:**  9.05±1.16  **After transfer:**  9.39±1.67 | **NM** | **Before transfer:**  0.0% had **severe hypoglycaemia.**  **After transfer:**  16 % had **severe hypoglycaemia.** | **NM** | **Before transfer:**  **Diabetes knowledge (mean)**  11.81±1.20  **Diabetes empowerment (mean)**  33.62±5.61  **Global well-being (24h) (mean)**  67.08±26.06  **Global well-being (one month) (mean)**  64.04±26.30  **Life satisfaction (mean)**  21.77±6.60  **Perceived stress (mean)**  47.96±10.86  **Depression**  6.73±6.21  **After transfer:**  **Diabetes knowledge (mean)**  11.62 ±1.36  **Diabetes empowerment (mean)**  34.32 ± 6.24  **Global well-being (24h) (mean)**  82.43 ± 18.96  **Global well-being (one month) (mean)**  82.41±16.97  **Life satisfaction (mean)**  26.22±6.69  **Perceived stress (mean)**  39.49±9.32  **Depression (mean)**  4.57 ±4.66" |
| 9 | **Average HbA1C (%)**  **Before transfer:**  10.2  **After transfer:**  8.3  P-value 0.01 | **At baseline:**  **Transfer completion**  11 of 14  **Retention to adult care originally referred to.**  9 of 11  **Time to transfer.**  15 weeks  **Adult dm visit per annum.**  3  **At 12 months:**  **Transfer completion**  12 of 12 P=0.2  **Retention to adult care originally referred to.**  10 of 12 p=1.0  **Time to transfer.**  14 weeks p= 0.7  **Adult dm visit per annum.**  2 P=0.4 | **At baseline:**  **Low range** **microalbuminuria**  2  More than 1 **recurrence of** **DM related hospitalization**  1  **Non-proliferative diabetic retinopathy**  1  **At 12 months:**  **Non-proliferative diabetic retinopathy**  3  P= 0.6 | **NM** | **NM** |
| 10 | **HbA1C % (mean)**  **At transition:**  8.3% (CI 7.9-8.7)  **After 1 year:**  7.4% (CI 6.9-7.9)  **After 2 years:**  7.4% (CI 6.9-7.4) | **After 1 year:**  **Clinic attendance**  68 % | **After 1 year:**  **DKA**  2 events (2 people)  **Severe hypoglycaemia events.**  6 | **NM** | **After 1 year:**  **Disease impact**  44.2±6.3  **Disease-related worries**  16.1±3.4  **Life satisfaction**  66.6±12.8  **At transition:**  **Disease impact**  46.3±9  **Disease-related worries**  18.4±4.7  **Life satisfaction**  59.5±14.6 |
| 11 | **NM** | **Patient activation measure 13 (PAM13-D) (mean ±SD)**  **At baseline**  78.40±2.41  **At 6 months**  75.85±14.89 | **NM** | **Health-related transition competence scale**  **At baseline**  53.57±5.83  **At 6 months**  49.49±7.12 | **NM** |
| 12 | **HbA1C % (mean)**  **At baseline:**  9.7% ±2.38  **At 6 months:**  9.0% ±1.88  p value <.001 | **NM** | **NM** | **Blood Glucose Monitoring Frequency checks per day**  **At baseline:**  2.5 ± 1.94  **At 6 months:**  3.5 ± 1.62  p value <.001 | **NM** |
| 13 | **HbA1C % (average)**  **Before transition:**  8.7%  **After transition:**  8.9% | **Paediatric people referred to adult care (%)**  **Before transition:**  11% of 191  **After transition:**  30% of 180  **Paediatric people who attended an adult visit (%)**  **Before transition:**  10.5% of 191  p=0.0001  **After transition:**  27.8% of 180  p=0.0001 | **Average number of DM-related ED visits during the transition gap (number)**  **After transition:**  0.17±0.47 | **NM** | **NM** |
| 14 | **HbA1C % (mean)**  **At 12 months:**  8·4±1·9  **At 24 months:**  8·4±1·7 | **Number of clinic appointments attended (12 months VS 24 months)**  2·3±1·1 VS 2·5±1·3  **Disengaged (12 months VS 24 months)**  6% VS 6% | **NM** | **NM** | **NM** |
| 15 | **HbA1C % (mean)**  **Before transition:**  9.5±1.8%  **After transition:**  10.49±1.68%  P<0.001 | **After transition:**  **Gap 48% less than 6 months.**  **After transition:**  **attendance: 80%** | **DM related hospitalisations**  **Before transition:**  48%  **After transition:**  59% | **NM** | **NM** |
| 16 | **HbA1C**  **At 1st appointment:**  8.5% (IQR= 4.1-17.2)  **At 18 months:**  8.6% (IQR= 7.7-10.2)  **At 30 months:**  8.7% (IQR= 7.7-10) | **NM** | **Mean Length of Stay of DKA admission stays.**  **in 2001:**  6.56 days  **in 2014:**  2.36 days | **NM** | **NM** |
| 17 | **NM** | **Autonomy in appointments (Median-IQR)**  **At baseline**  9.00 - 7.00, 1.00  **At final visit:**  12.00 - 9.00, 14.00 P=<0.001 | **NM** | **NM** | **NM** |
| 18 | **HbA1c (average)**  **Before transition:**  9.5%  **After transition:**  8.9% | **Clinic non-attendance rate.**  **Before transition:**  19.80%  **After transition:**  15.5% | **Diabetes-related hospital admission**  **Before transition:**  34.7%  **After transition:**  16.9% | **NM** | **NM** |
| 19 | **HbA1C (mean)**  **Before transition:**  **NR**  **After transition:**  7.9 ± 1.7% | **Rate of 1^st^ and second adult clinic attendance.**  **Before transition:**  14 (100%)  **After transition:**  6 (46%)  P value 0.001 | **Number of DKA hospitalisation.**  **Before transition:**  3  **After transition:**  0  P value 0.06 | **NM** | **NM** |
| 20 | **HbA1C (mean)**  **At 18 months:**  8.63% (1.49)  p value 0.758  **At 12 months after completion:**  8.80% (1.55)  P value 0.057 | **Clinic visit:**  **Before transition:**  3.6±1.2  **After transition:**  4.1 ±1.1 | **NM** | **NM** | **Diabetes distress and impact of diabetes on quality of life.**  **At 18 months:**  66.3±9.4  **At 12 months after completion:**  65.8±9.4  **At 18 months:**  68.2±9.5  **At 12 months after completion:**  65.5 (10.8) |
| 21 | **HbA1C (mean)**  **Before transition:**  8.3±1.4  **After transition:**  8.2±1.4  p 0.48 | **NM** | **Before transition:**  **<2 hypoglycaemia episodes/week**  140±62.2  **>5 hypoglycaemia episodes/week**  15±6.9  **Severe hypoglycaemia episodes/patient/year**  0.23±0.64  **After transition:**  **<2 hypoglycaemia episodes/week**  64±28.4  p 0.001  **>5 hypoglycaemia episodes/week**  9±3.9  P 0.09  **Severe hypoglycaemia episodes/patient/year**  0.05±0.34  p 0.001  **Lipodystrophy**  60.9% (20.3% severe) | **NM** | **NR** |
| 22 | **HbA1C (mean)**  **Before transition:**  **At 12 months:**  9.2% ± 1.8  **At 18 months:**  9.2% ± 1.9  P value 0.2  **After transition:**  **At 12 months:**  8.8% ± 1.9  **At 18 months:**  8.8% ± 1.9 | **Before transition:**  **Non-attendance:**  **At 12 months:**  47.1%  **At 18 months:**  47.1%  P value < 0.01  **follow up visits.**  **At 12 months:**  3.0 ± 5.9  **At 18 months:**  3.0 ± 5.9  P value < 0.01  **After transition:**  **Non-attendance:**  **At 12 months:**  11.9%  **At 18 months:**  9.9%  **follow up visits.**  **At 12 months:**  5.8 ± 6.0  **18 months:**  6.0 ± 6.0 | **Before transition:**  **ED visits:**  **At 12 months:**  0.2 ± 0.5  **At 18 Months:**  0.2 ± 0.5  P value 0.2  **DKA episodes:**  **At 12 months:**  0.1± 0.3  **At 18 months:**  0.1± 0.3  P value 0.1  **After transition:**  **ED visits:**  **At 12 months:**  0.4 ± 1.4  **At 18 months:**  0.5 ± 1.4  **DKA episodes:**  **At 12 months:**  0.1 ± 0.3  **At 18 months:**  0.1 ± 0.3 | **NM** | **NM** |
| 23 | **HbA1C (mean)**  8.35 ± 1.28 | **NM** | **Participants n=222**  **Diabetic retinopathy (%)**  **Background**  57 (25.7)  **Laser-treated**  6 (2.7)  **Diabetic nephropathy (%)**  108 (95.6)  **Microalbuminuria**  7 (3.2)  **Proteinuria**  1 (0.5) | **NM** | **Participants n=222**  **Disorders of eating**  **Behaviours (%)**  17 (7.7)  **Depressive and/or**  **Anxiety (%)**  24 (10.8)  **Fear of hypoglycemia (%)**  12 (5.4)  **Poor diabetes acceptance (%)**  37 (16.7) |
| 24 | **Pre-transfer HbA1c (mmol/mol) (%)**  **Control**  86.57±39.40  10.1±5.8  **Intervention**  77.08±32.98  9.2±5.2  p=0.547  **Post-transfer HbA1c (mmol/mol)**  **(%)**  **Control**  95.78±42.7  10.9±6.1  **Intervention**  78.50±30.00  9.3±4.9  p=0.106 | **NM** | **Pre-transfer total cholesterol (mmol/L)** 4.48±0.96 4.70±0.98 p=0.305  **Post-transfer total cholesterol (mmol/L)** 4.58±1.09 4.81±1.02 p=0.350  **Pre-transfer non-HDL cholesterol (mmol/L)** 3.10±1.12 3.05±1.1 p=0.813  **Post-transfer non-HDL cholesterol (mmol/L)** 3.13±1.20 2.74±1.29 p=0.164 | **NM** | **NM** |
